# Supplementary material for: Different soluble aggregates of Aβ42 can give rise to cellular toxicity through different mechanisms
Source: Nat Commun. 2019 Apr 4;10:1541. doi: 10.1038/s41467-019-09477-3 (PMC6449370; doi:10.1038/s41467-019-09477-3)
Supplement: Supplementary file 1 — Supplementary Information File [file 41467_2019_9477_MOESM1_ESM.pdf]

Supplementary Information for

Different soluble aggregates of Aβ42 can give rise to cellular toxicity through different mechanisms

De et al.

Supplementary Figures

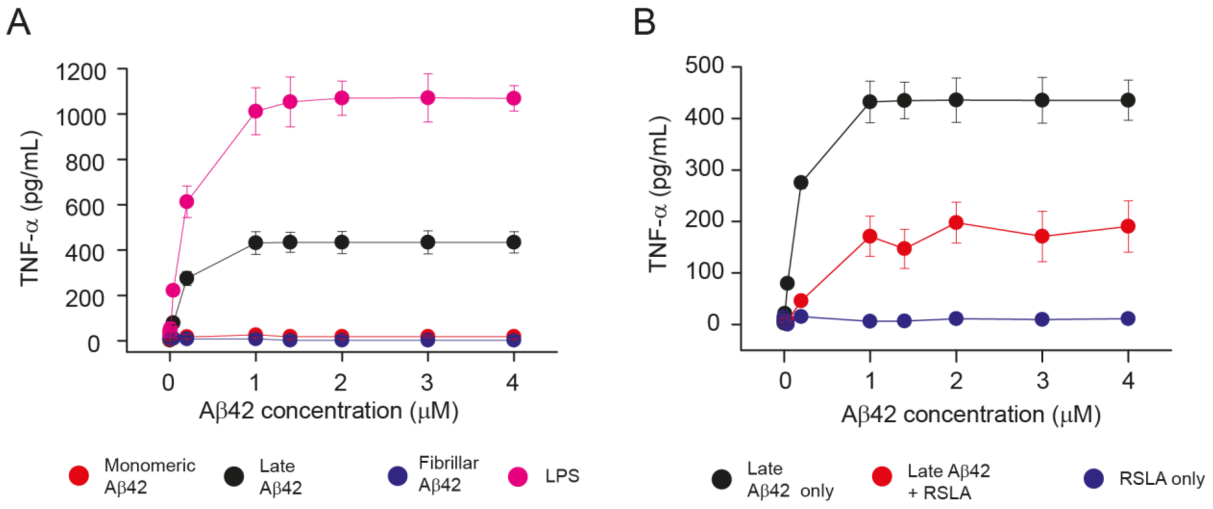

Supplementary Figure 1. (A) Soluble aggregates, but not monomeric and fibrillar forms, of Aβ42 induce inflammation. TNF-α production by BV2 cells[26] in response to sustained exposure over 24 hours to monomeric, aggregated and fibrillar forms of Aβ42, ranging in concentration from 0.001 to 4 µM (total monomer concentration) and control LPS. **(B) RSLA blocks Aβ42 aggregate induced inflammation.** 100 ng/ml of TLR4 antagonist RSLA was incubated with and without aggregated Aβ42 over the same period of time (n=5, error bars are the standard error of the mean)

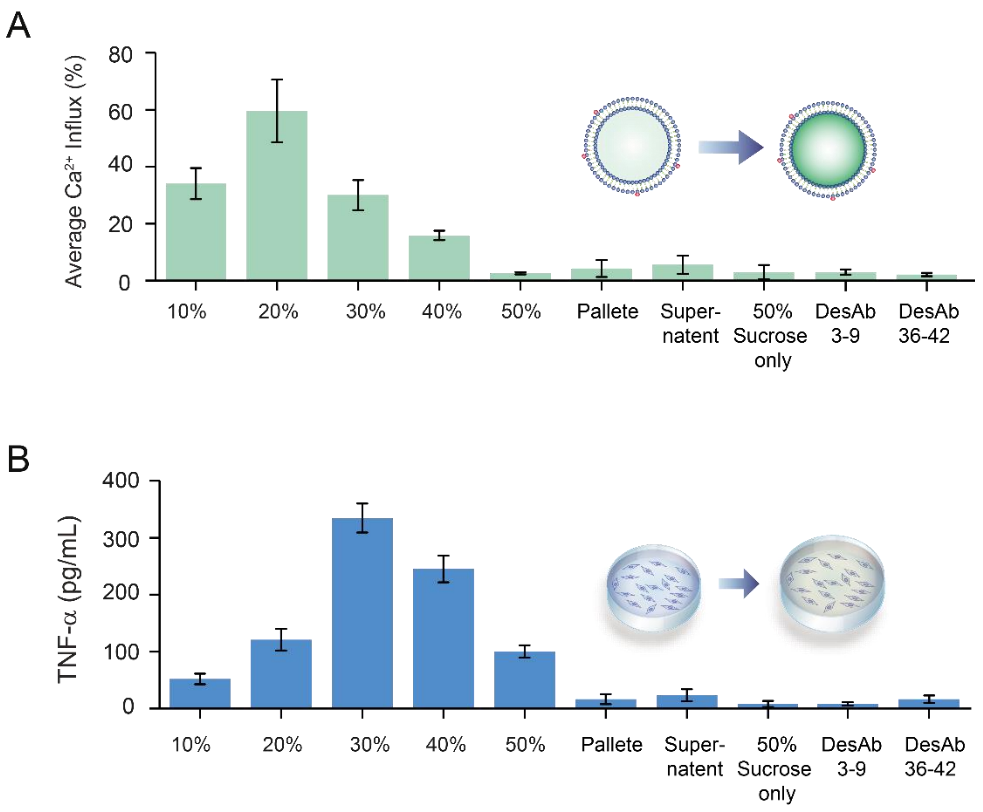

Supplementary Figure 2. Soluble Aβ42 aggregates of different sizes exhibit different relative toxicity. Comparison of the toxicity of soluble aggregates present at different sucrose fractions using: **(A)** membrane permeabilization, and **(B)** inflammatory response assays. Neither supernatant, pellet, sucrose solution or antibodies induce significant membrane permeation or inflammation. The sucrose fractions were diluted 1: 10 buffer for all the experiments. These experiments were carried out for two independent aggregation reactions of Aβ42 (n=2) and the error bars represent the standard deviation of the mean for each field of view.

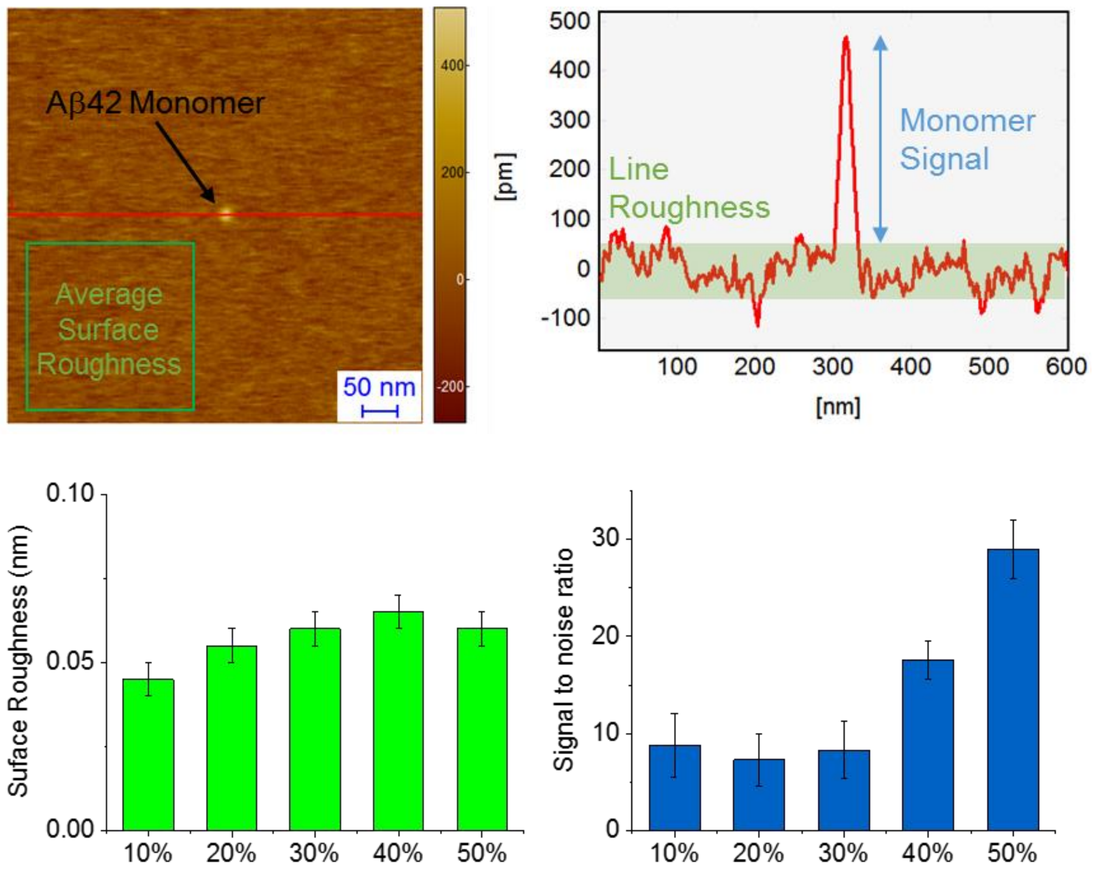

Supplementary Figure 3. Determination of signal to noise ratio of the AFM maps. Measurements enabled imaging of objects as small as individual Aβ42 monomers. We calculated the signal to noise ratio for each sample, defined as the average height of the aggregates in each map (signal) and the roughness of the surface (noise). The average level of noise is well below 1 angstrom and the signal to noise ratio for measuring a single monomer is close to 10 and reaches 30 for intermediate oligomeric species. Thus, the characterisation of cross-sectional height can be performed with high sensitivity and accuracy.

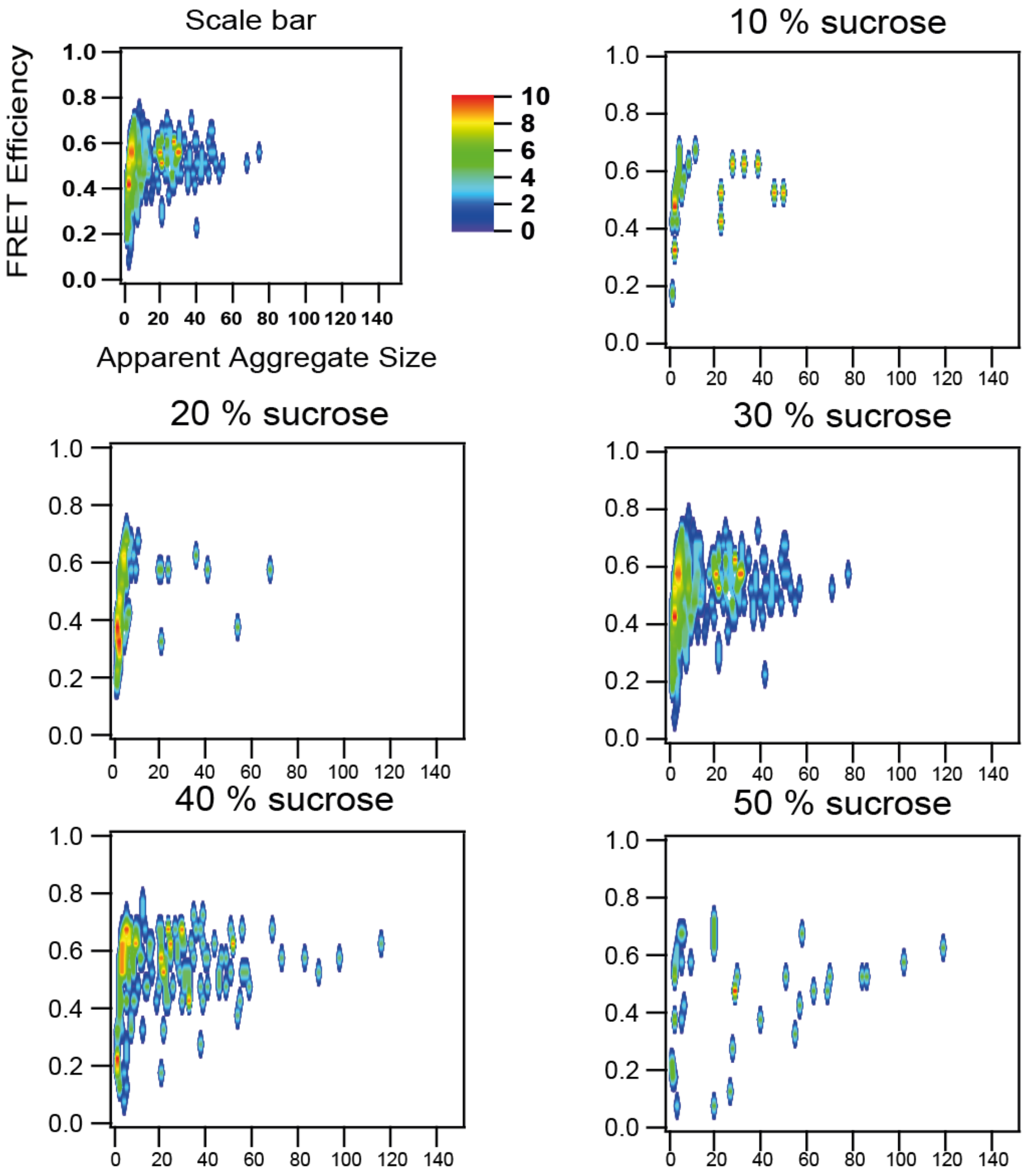

Supplementary Figure 4 Analysis of dual-labelled co-aggregates as measured with the single molecule FRET. The FRET efficiency is plotted against the apparent oligomer size inferred from the intensity. The aggregates increase in size and the FRET efficiency shifts to higher values. The FRET histograms are representative of the gradient centrifugation fractions and show all aggregates plotted of a single measurement. A single measurement of each fraction contains usually more than 10 000 aggregates, if not the concentrations were adjusted to do so.

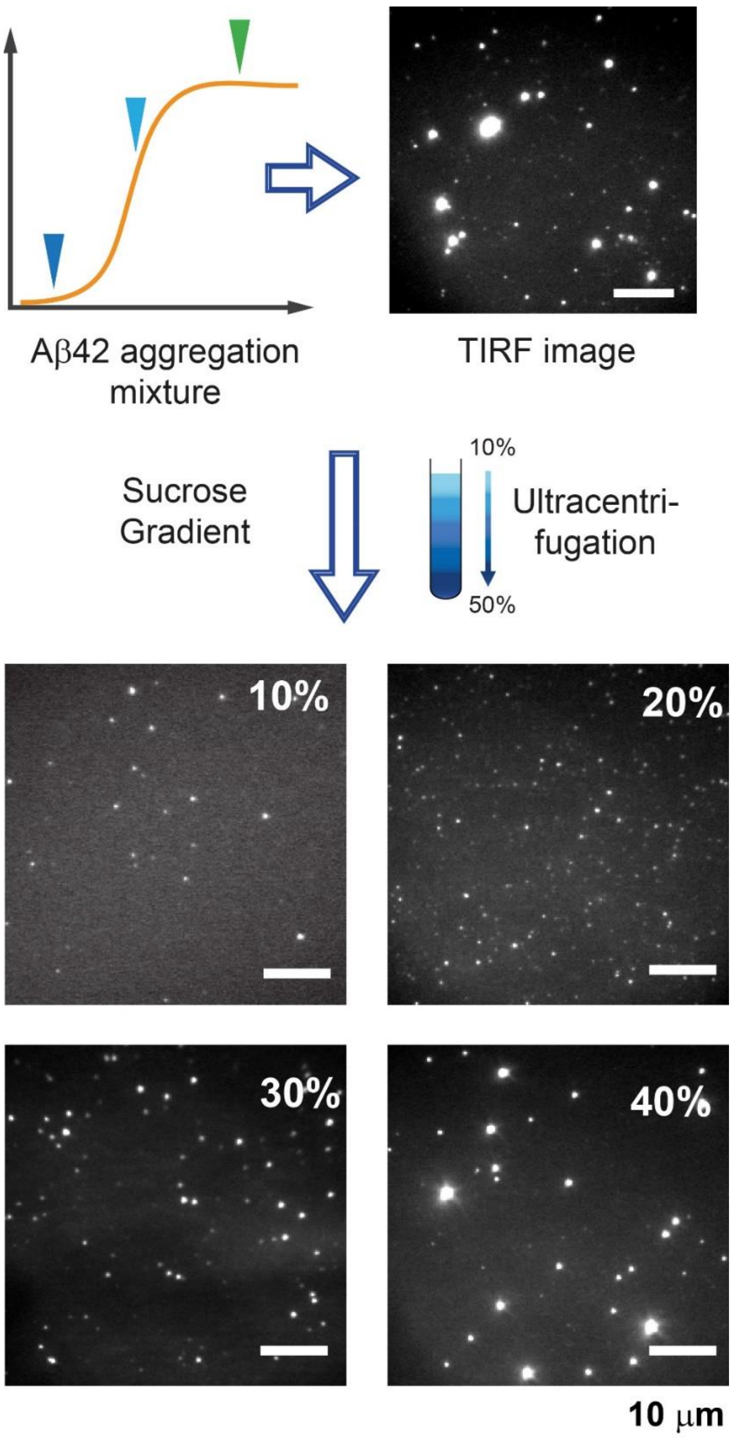

Supplementary Figure 5. Single aggregate imaging using Total internal reflection fluorescence imaging. This aggregated mixture of Aβ42 collected at three different time points and was imaged (input) using TIRF in single aggregate level. The input was then loaded onto a sucrose step-gradient that is divided into fractions, and was subsequently imaged using TIRF microscopy.

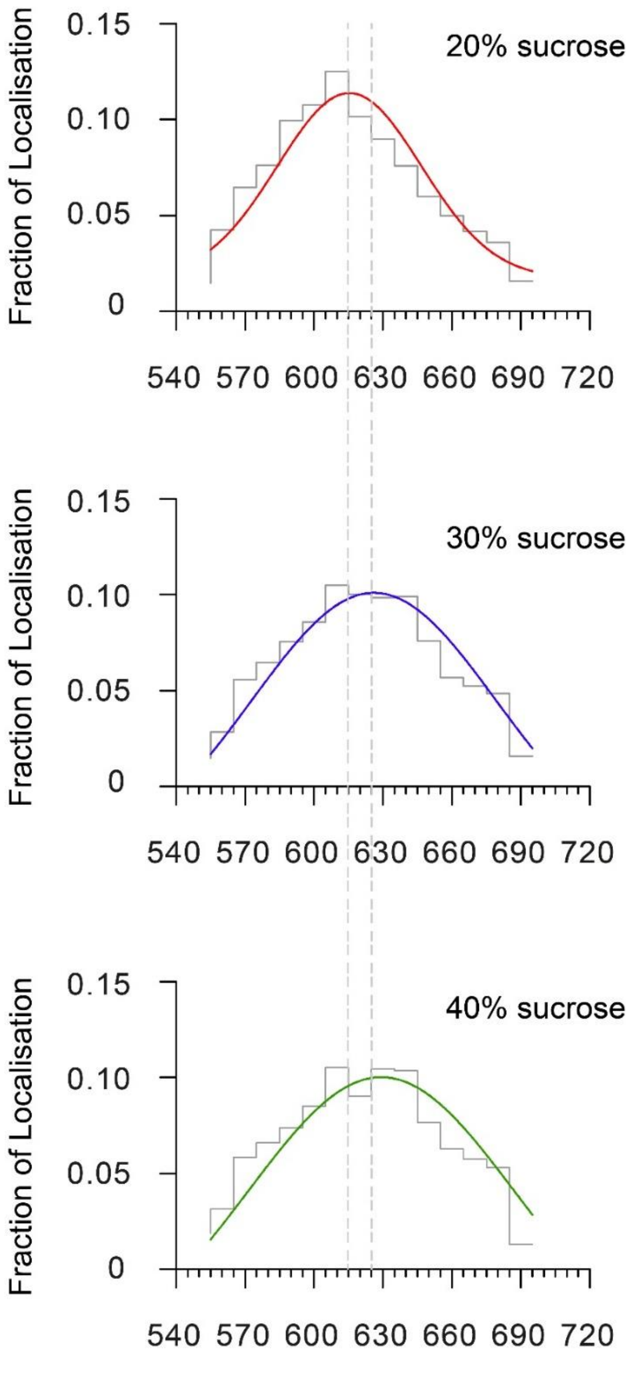

Supplementary Figure 6. sPAINT imaging of the surface hydrophobicity of the protein aggregates present in the different sucrose fractions. Frequency histogram of the sPAINT emission maxima from the individual protein aggregates present at 20%, 30% and 40% sucrose solution (Total no of aggregate analysed 106, 145 and 67 for 20% 30% and 40% sucrose fraction respectively). Each fraction is diluted to 50 times before imaging using sPAINT. Aggregate clumping prevented measurements being made on the 50% fraction.

Each distribution was fitted with a Gaussian function The peak for 20% fraction shows a blue shift of approximately 10 nm compared to the 30% and 40% sucrose fractions.

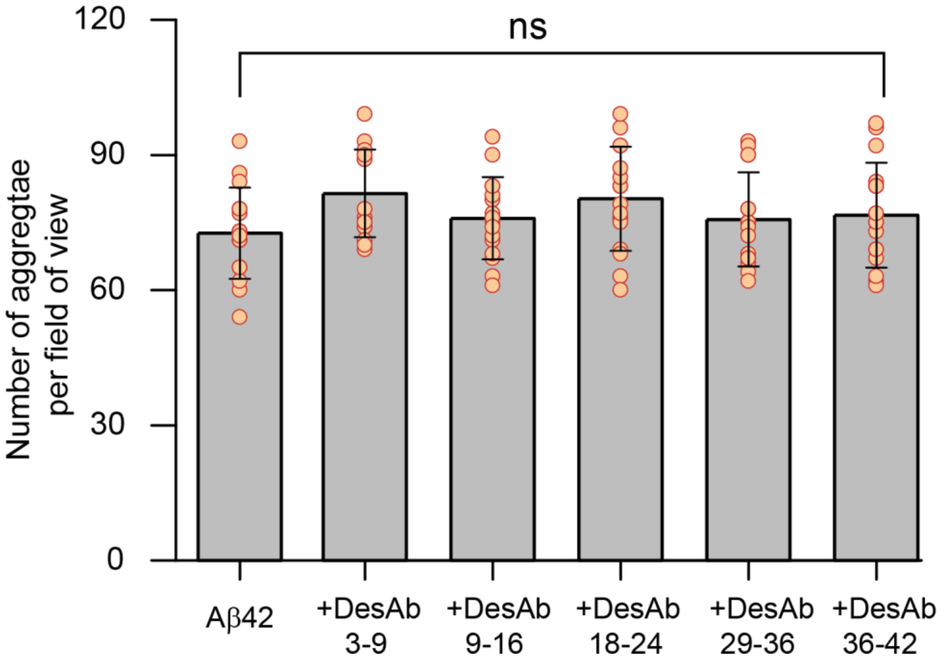

Supplementary Figure 7. Effects of designed antibodies on the stability of ThT-positive Aβ42 aggregates. An aggregated solution of 1 μM total Aβ42 monomer concentration was incubated with 150 nM of each single chain antibody for 60 min and aggregates were counted as described previously[35]. There is no significant difference in the number of ThT-active species before and after the addition of the designed antibodies indicating that the addition of these antibodies does not lead to the disaggregation of the aggregates within the time scale of our experiments. (error bars represent the standard deviation, two sample unpaired t-test).

Supplementary Table

Antibody	Primer
**DesAb3-9**	FOR 5' P-TGCGCGAGGAAGAGGCGGCCGC 3'<br>REV 5' P-GGGTCAGGGTTTCATGAGATCCCGCTGCGCAAT 3'
**DesAb13-19**	FOR 5' P-GTGATTAAAGAAATTGAGGAAGAGGCGGCCGC 3'<br>REV 5' P-GCTCAGAGATCCCGCTGCGCAAT 3'
**DesAb18-24**	FOR 5' P-CGAAGCGGAGGAAGAGGCGGCCGC 3'<br>REV 5' P-GTGCCCACAAACACAGATCCCGCTGCGCAAT 3'
**DesAb29-36**	FOR 5' P-GCGACCGTGGAGGAAGAGGCGGCCGC 3'<br>REV 5' P-TTTATACATGCTGCCAGATCCCGCTGCGCAAT 3'
**DesAb36-42**	FOR 5' P-CGGAACTGGAGGAAGAGGCGGCCGC 3'<br>REV 5' P-CTTTAATGCCCAGAGATCCCGCTGCGCAAT 3'

Supplementary Table1: DesAbs were generated using PCR with the following 5' phosphorylated primers (P= phosphate group)
